# Yucatan Minipig Knee Meniscus Regional Biomechanics and Biochemical Structure Support its Suitability as a Large Animal Model for Translational Research

**DOI:** 10.3389/fbioe.2022.844416

**Published:** 2022-02-21

**Authors:** Erik A. Gonzalez-Leon, Jerry C. Hu, Kyriacos A. Athanasiou

**Affiliations:** Department of Biomedical Engineering, University of California, Irvine, CA, United States

**Keywords:** Yucatan minipig, knee meniscus, regenerative medicine, biomechanics, preclinical animal model

## Abstract

Knee meniscus injuries are the most frequent causes of orthopedic surgical procedures in the U.S., motivating tissue engineering attempts and the need for suitable animal models. Despite extensive use in cardiovascular research and the existence of characterization data for the menisci of farm pigs, the farm pig may not be a desirable preclinical model for the meniscus due to rapid weight gain. Minipigs are conducive to *in vivo* experiments due to their slower growth rate than farm pigs and similarity in weight to humans. However, characterization of minipig knee menisci is lacking**.** The objective of this study was to extensively characterize structural and functional properties within different regions of both medial and lateral Yucatan minipig knee menisci to inform this model’s suitability as a preclinical model for meniscal therapies. Menisci measured 23.2–24.8 mm in anteroposterior length (33–40 mm for human), 7.7–11.4 mm in width (8.3–14.8 mm for human), and 6.4–8.4 mm in peripheral height (5–7 mm for human). Per wet weight, biochemical evaluation revealed 23.9–31.3% collagen (COL; 22% for human) and 1.20–2.57% glycosaminoglycans (GAG; 0.8% for human). Also, per dry weight, pyridinoline crosslinks (PYR) were 0.12–0.16% (0.12% for human) and, when normalized to collagen content, reached as high as 1.45–1.96 ng/µg. Biomechanical testing revealed circumferential Young’s modulus of 78.4–116.2 MPa (100–300 MPa for human), circumferential ultimate tensile strength (UTS) of 18.2–25.9 MPa (12–18 MPa for human), radial Young’s modulus of 2.5–10.9 MPa (10–30 MPa for human), radial UTS of 2.5–4.2 MPa (1–4 MPa for human), aggregate modulus of 157–287 kPa (100–150 kPa for human), and shear modulus of 91–147 kPa (120 kPa for human). Anisotropy indices ranged from 11.2–49.4 and 6.3–11.2 for tensile stiffness and strength (approximately 10 for human), respectively. Regional differences in mechanical and biochemical properties within the minipig medial meniscus were observed; specifically, GAG, PYR, PYR/COL, radial stiffness, and Young’s modulus anisotropy varied by region. The posterior region of the medial meniscus exhibited the lowest radial stiffness, which is also seen in humans and corresponds to the most prevalent location for meniscal lesions. Overall, similarities between minipig and human menisci support the use of minipigs for meniscus translational research.

## Introduction

Damage to the knee meniscus can result from trauma or age-related degeneration; meniscal lesions are the most common intra-articular knee injury and account for the most frequent cause of orthopedic surgical procedures in the U.S. (Salata et al., 2010). Specifically, up to 20% of orthopedic procedures involve surgery on the meniscus, leading to approximately 850,00 patients per year (Logerstedt et al., 2010). The medial meniscus is about 4-times more likely to be damaged and undergo surgery compared to the lateral meniscus (Baker et al., 1985). Additionally, the meniscus is a fibrocartilaginous tissue that is nearly avascular and, thus, is generally not amenable to repair. Differences in injury prevalence between medial and lateral menisci can result from differences in structural properties and, thus, functionality, making it important to consider these properties during every step of developing new therapies, such as tissue engineered menisci.

Options for the management of meniscal injuries vary with respect to disease severity and type, ranging from physical therapy to invasive surgical intervention (Herrlin et al., 2007; Maffulli et al., 2010; Katz et al., 2013). Meniscectomy, the partial or complete removal of the knee meniscus, can relieve pain but is reserved for cases in which meniscus repair is unlikely (e.g., tears in the avascular portion) (Barber-Westin and Noyes, 2014; Xu and Zhao, 2015). Removal of either meniscus greatly predisposes a patient to osteoarthritis (Rangger et al., 1997). Thus, novel regenerative solutions for knee meniscus repair and replacement are required. Toward demonstrating efficacy of novel meniscal therapies, appropriate animal models will be needed to traverse the regulatory process. These animals should have menisci with morphological, biomechanical, and biochemical properties that are comparable to humans; similarities in gait, joint anatomy, and joint biomechanics should also be considered to facilitate translation (Donahue et al., 2019).

Engineered meniscal tissues are expected to experience complex loading patterns within the knee. For example, human medial menisci have been shown to have mechanical properties that vary by topographical location (Sweigart et al., 2004). Additionally, knee menisci have anisotropic tensile properties, or different mechanical properties when tested in circumferential versus radial directions; this difference in mechanical properties stems from circumferentially aligned collagen fibers that convert compressive forces into tensile hoop stresses. It is posited that tissue engineered implants should closely resemble the native tissue toward restoring function *in vivo*; thus, acquisition of complete design parameters from native tissue is crucial. Furthermore, engineered implants would require testing in a large animal model to show safety and efficacy prior to human trials (Donahue et al., 2019). Although characterization studies of human and farm pig knee menisci have been conducted (Sweigart et al., 2004; Sweigart and Athanasiou, 2005; Takroni et al., 2016) and bovine cells have been used to tissue engineer menisci (Huey and Athanasiou, 2011; Hadidi and Athanasiou, 2013; Gonzalez-Leon et al., 2020), neither farm pig nor bovine models may be suitable for preclinical testing due to these animals being vastly different from humans in terms of weight. More frequently, animal models such as the goat, sheep, dog, and rabbit have been used for meniscus studies (Chevrier et al., 2009; Deponti et al., 2015; Bansal et al., 2017; Brzezinski et al., 2017). An emerging large animal model is the minipig, which has been proposed as a possible model that can be incorporated into future guidance documents for meniscus repair (Donahue et al., 2019), but data on the knee menisci of minipigs are lacking.

The minipig model, specifically the Yucatan breed, is often used in biomedical research (Khoshnevis et al., 2017, 2020; Poupin et al., 2019; Melnick et al., 2020) and has been gaining popularity in orthopedic research and musculoskeletal science (Cone et al., 2017; Bansal et al., 2020; Kremen et al., 2020; Nordberg et al., 2021). Yucatan minipigs share physiological similarities with humans. For example, minipig neural vascularization patterns, central nervous system physiology, and weights are comparable to humans (Vodička et al., 2005; Walpole et al., 2012; Moriguchi et al., 2013; Kim et al., 2015); additionally, adult pig menisci have been shown to have similar vascularization patterns to humans (Peretti et al., 2004). In contrast to farm pigs, minipigs are more suitable for long-term studies because their smaller size leads to reductions in needs related to handling, housing, surgery, anesthesia, and food (Mardas et al., 2014). Particularly important is that the minipig weight gain throughout a study is not as drastic as farm pigs. For example, a Yucatan minipig weighs approximately 20–30 kg at sexual maturity (5–6 months old) and has a typical growth rate of 3–5 kg per month, while Yorkshire/Landrace hybrid pigs at sexual maturity (5–6 months) weigh well over 100 kg and continue to grow at 10–20 kg per month (Swine Models Premier BioSource). Because the Yucatan minipig provides physiological similarities to humans, requires less resources for surgery and handling, and change less over a study’s period as compared to farm pigs, its potential as a large animal model for meniscus research should be investigated, particularly through characterization of morphological, mechanical, and biochemical properties of native tissue.

This work characterized the medial and lateral knee menisci of Yucatan minipigs through extensive analyses of structure-function relationships within the native tissue. Minipig knee menisci were investigated by gross morphology, histology, mechanical testing under tension and compression, and biochemical analyses. Furthermore, motivated by topographical differences in properties in human menisci, different regions of the minipig menisci were examined for mechanical anisotropy and degree of collagen crosslinking to provide greater insight on the native tissue’s function. Because skeletally mature minipigs are similar in weight to humans and because regional differences in mechanical properties have been observed in human menisci, we hypothesized that 1) gross morphological dimensions of minipig menisci would fall within human menisci ranges, 2) as with humans, regional differences in mechanical properties would be observed in the minipig menisci, and 3) regional differences in mechanical properties would correspond to differences in collagen, glycosaminoglycan (GAG), and crosslink content. The data here will serve to advance our understanding of the regional structure-function relationships of minipig knee menisci, to provide benchmarks to assist the creation of novel regenerative solutions for human meniscal lesions, and to provide critical information regarding the suitability of the minipig as a model for investigations of the knee meniscus.

## Materials and Methods

### Animals, Knee Meniscus Gross Morphology, Histology, and Macroscopic Characterization

**FIGURE 1:**
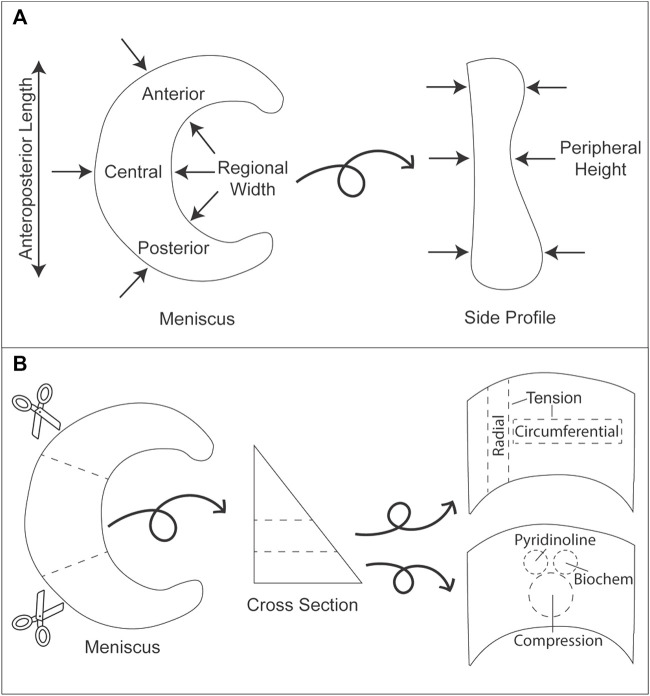
Gross morphology measurements and division of minipig knee menisci for mechanical and biochemical analyses. **(A)** Arrows indicate the locations where measurements were taken for anteroposterior length, regional width, and peripheral height. **(B)** Each meniscus was cut into three regions (anterior, central, posterior). Subsequently, each section was cut into layers from which tensile (circumferential and radial directions), compressive (creep indentation), biochemistry, and mass spectrometry samples were collected.

**FIGURE 2:**
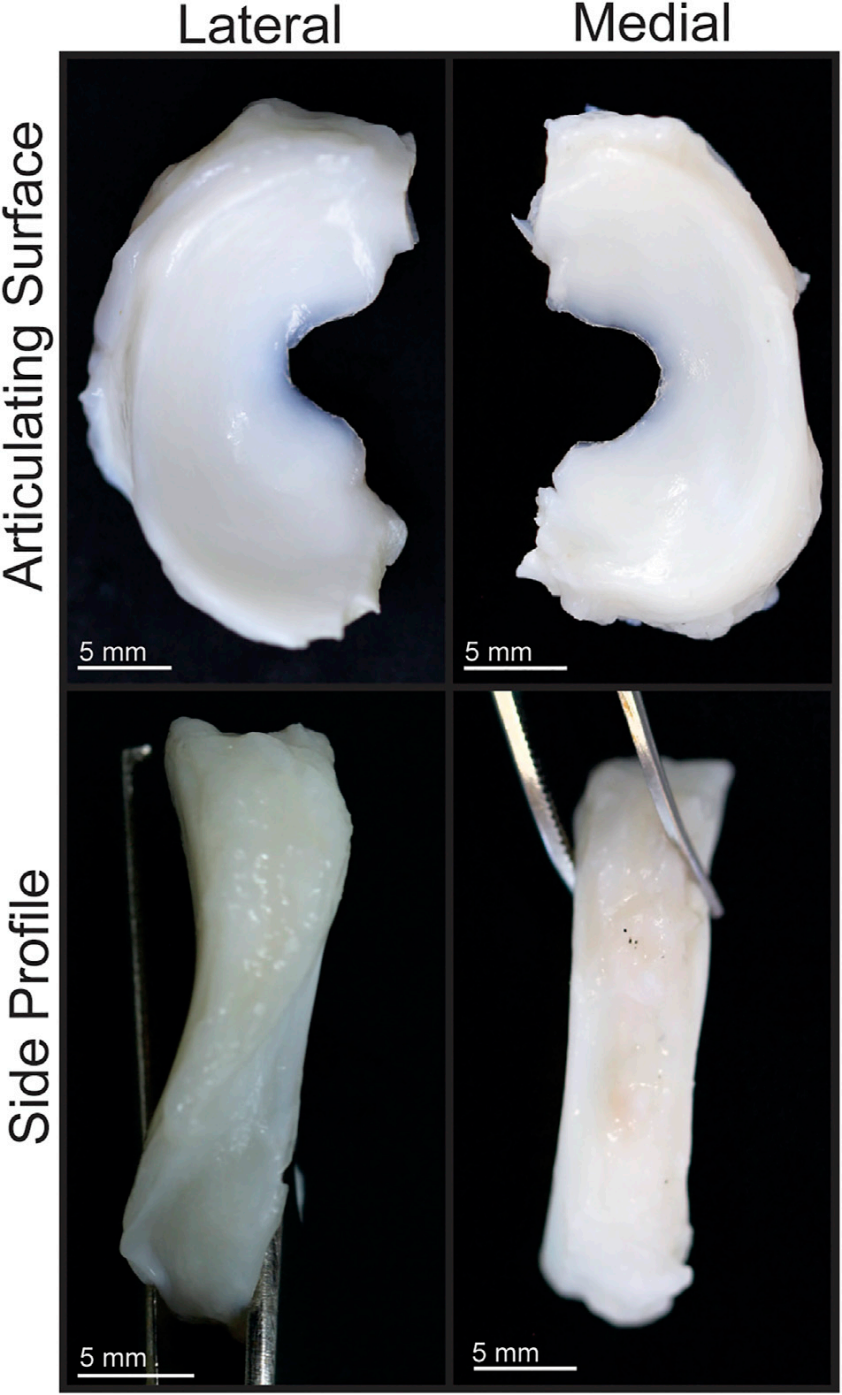
Gross morphology of Yucatan minipig knee menisci. Articulating surfaces and side profiles of medial and lateral menisci are shown.

**FIGURE 3:**
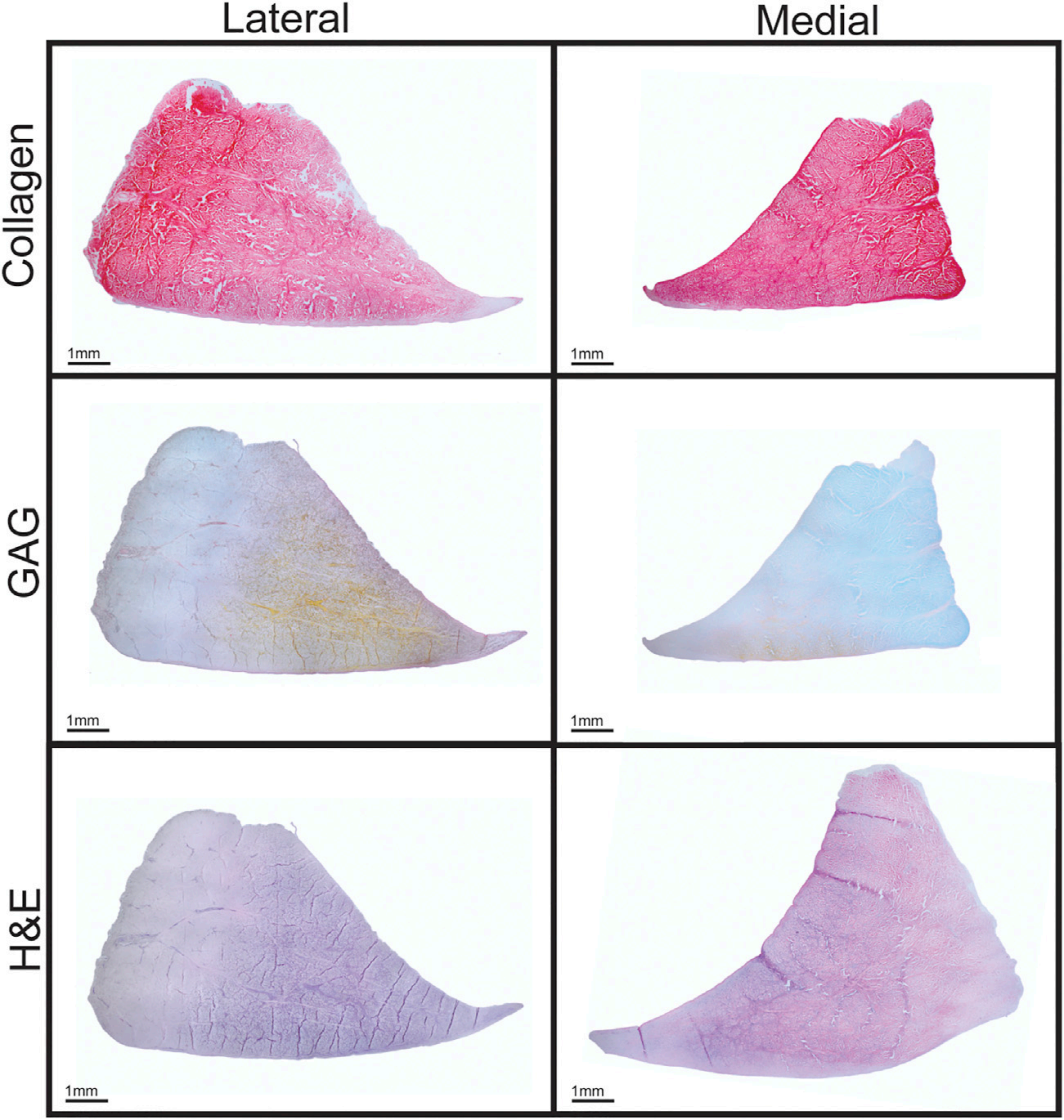
Histological staining of Yucatan minipig knee menisci. Cross sections of menisci stained for collagen (picrosirius red), GAG (Safranin-O), and cell content (H&E) are shown.

Knee menisci were obtained from eight healthy, skeletally mature, 16–18-month-old male and female Yucatan minipigs that were sacrificed due to reasons unrelated to this study. The menisci were excised and subsequently frozen in PBS-containing protease inhibitors 10 mmol/L N-ethylmaleimide and 1 mmol/L phenylmethylsufonyl fluoride (Sigma) at −20°C. Menisci were thawed and photographed, and the dimensions were measured using ImageJ (NIH; Figures 1A,2) before dividing each meniscus into three regions (anterior, central, posterior). Pieces for mechanical testing and biochemical analysis were resected from the white-red zone of each region (Figure 1B), while histology samples comprised of a cross section taken from the central region of each meniscus. For histology, construct samples were fixed in 10% neutral buffered formalin, then embedded in paraffin and sectioned at 5 μm. Safranin-O/fast green, picrosirius red, and hematoxylin and eosin (H&E) stains were conducted to visualize GAG, collagen, and cell distributions, respectively (Figure 3).

### Tensile and Compressive Testing

Tensile properties were assessed using uniaxial, strain-to-failure testing in circumferential and radial directions. Samples were cut into rectangular strips and photographed, and the dimensions were measured with ImageJ. Samples were then clamped within a uniaxial testing machine (Instron model 5,565) and subjected to a 1% s^−1^ strain rate until failure. Young’s modulus (E_Y_) was calculated from the linear portion of the stress-strain curve, and ultimate tensile strength (UTS) was calculated from the maximum stress.

Compressive properties were assessed via creep indentation testing of punches measuring 3 mm in diameter and placed into an automated indentation machine while submerged in phosphate buffered saline (PBS), as previously described (Brown et al., 2018; Espinosa et al., 2021b). Briefly, tissue punches were tested using a 0.5 mN tare load followed by a 0.04–0.05 N test load to maintain ∼10% applied strain. The loads were applied to the surface of specimens through a 1.0 mm diameter, flat-ended, porous tip, perpendicular to the surface at the center of the sample. The sample surface is assumed to be a semi-infinite half space, which allows the single measurement point to be representative of the whole sample. The tissue was allowed to reach creep equilibrium while the deformation was recorded over time. Using the analytical solution for the axisymmetric Boussinesq problem with Papkovich potential functions, preliminary estimations of the aggregate modulus of the samples were obtained. Using the linear biphasic theory followed by a finite element model, intrinsic biomechanical properties of the samples such as aggregate modulus, shear modulus, Poisson’s ratio, and permeability were calculated (Athanasiou et al., 1995; Elder et al., 2010).

### Analysis of Tissue Biochemical Content

Biochemistry samples were weighed wet, then frozen and lyophilized to acquire dry weights. Collagen content was measured with the use of a Sircol standard (Biocolor) and a modified chloramine-T colorimetric hydroxyproline assay (Cissell et al., 2017). GAG content was quantified using the Blyscan Glycosaminoglycan assay kit (Biocolor). All quantification measurements for collagen and GAG content were performed with a GENios spectrophotometer/spectrofluorometer (TECAN).

Quantification of pyridinoline crosslink content was performed via a liquid chromatography mass spectrometry (LC-MS) assay (Naffa et al., 2019). Lyophilized samples were hydrolyzed in 6N HCl at 105°C for 18 h. After evaporation, dried hydrolysates were resuspended in 25% (v/v) acetonitrile and 0.1% (v/v) formic acid in water, centrifuged at 15,000 g for 5 min, and the supernatant was transferred to a LCMS autosampler vial. Liquid chromatography was carried out on a Cogent Diamond Hydride HPLC Column (2.1 mm × 150 mm, particle size 2.2 μm, pore size 120 Å, MicroSolv) and a pyridinoline standard (BOC Sciences) as previously described (Gonzalez-Leon et al., 2020).

### Statistical Analysis

For each biomechanical, biochemical, and morphological test, *n* = 5–7 samples were used. To identify outliers within groups, a ROUT test was applied using GraphPad Prism software; no outliers were identified. A Shapiro-Wilk test was applied using alpha = 0.01 to confirm that data within groups were normally distributed. Data were first analyzed using a Student’s *t*-test comparing aggregated data from all regions of the medial and of the lateral menisci to discern differences between the two sides. This level of analysis was motivated by literature showing that properties within medial and lateral menisci are different across multiple species. Next, a single factor analysis of variance (ANOVA) or Kruskal–Wallis test was used when appropriate to determine, for each meniscus, whether the properties differed by region; the levels consisted of anterior, central, and posterior regions. A Tukey’s HSD *post hoc* test was performed when merited. All statistics were performed with *p* < 0.05. All data are presented as means ± standard deviations. For all figures, a connecting letters report shows statistical significance as indicated by groups not sharing the same letters.

## Results

### Gross Morphology and Histology

The Yucatan minipig medial and lateral menisci were semi-lunar and wedge shaped (Figures 2, 3), with anteroposterior lengths of 23.2 and 24.8 mm, respectively; no significant difference in length was found between the two groups (Table 1). Significant difference was observed in width; medial meniscus width ranged from 7.7 to 10.2 mm across its regions while lateral meniscus width ranged from 8.4 to 11.4 mm. The posterior region was significantly wider than other regions for both menisci. Peripheral height also differed significantly; the medial and lateral meniscus peripheral heights varied from 6.4 to 6.6 mm and 6.4–8.4 mm, respectively. The anterior and posterior regions of the lateral meniscus exhibited significantly higher peripheral heights when compared to the central region; there were no significant differences in peripheral heights among medial meniscus regions.

**TABLE 1:** Morphological properties of minipig menisci. Student’s *t*-test showed a significant difference between medial and lateral menisci in hydration, width, and peripheral height values. For comparison of regions within each meniscus, Tukey’s HSD test showed significant differences among regions for both menisci in width, while the lateral meniscus exhibited differences in peripheral height values among its regions. Values marked with different letters within each category are significantly different among groups (*p* < 0.05), *n* = 7–8 per group. Human values of morphological properties from the literature are shown for comparison (Proffen et al., 2012; Takroni et al., 2016).

Meniscus	Region	Hydration (%)	Average hydration (%)	Antero-posterior Length (mm)	Width (mm)	Average Width (mm)	Peripheral height (mm)	Average Peripheral Height (mm)
Medial (Minipig)	Anterior	65.8 ± 2.7	64.0 ± 2.8^B^	23.2 ± 1.3	8.6 ± 0.8^B^	8.8 ± 0.9^B^	6.6 ± 0.7	6.5 ± 0.8^B^
	Central	62.3 ± 3.3			7.7 ± 0.9^B^		6.4 ± 1.0	
	Posterior	64.2 ± 1			10.2 ± 0.6^A^		6.6 ± 0.9	
Lateral (Minipig)	Anterior	68.5 ± 2.5	67.8 ± 3.5^A^	24.8 ± 2.4	9.4 ± 0.7^B^	9.7 ± 0.9^A^	7.9 ± 0.7^A^	7.5 ± 1.3^A^
	Central	67.8 ± 3.5			8.4 ± 0.9^B^		6.4 ± 0.8^B^	
	Posterior	67.1 ± 4.9			11.4 ± 1.1^A^		8.4 ± 1.4^A^	
Medial (Human)	Anterior	N/A	70–75	39.8 ± 3.7	8.5 ± 0.6	10.6 ± 0.8	5.5 ± 0.3	5.8 ± 0.3
	Central				8.3 ± 0.5		5.0 ± 0.5	
	Posterior				14.8 ± 0.8		7.0 ± 0.7	
Lateral (Human	Anterior	N/A		33.3 ± 3.5	11.5 ± 0.4	11.6 ± 0.2	6.4 ± 0.9	6.3 ± 0.4
	Central				11.6 ± 0.5		6.3 ± 0.5	
	Posterior				11.7 ± 0.3		6.2 ± 0.8	

### Tissue Biomechanics

**FIGURE 4:**
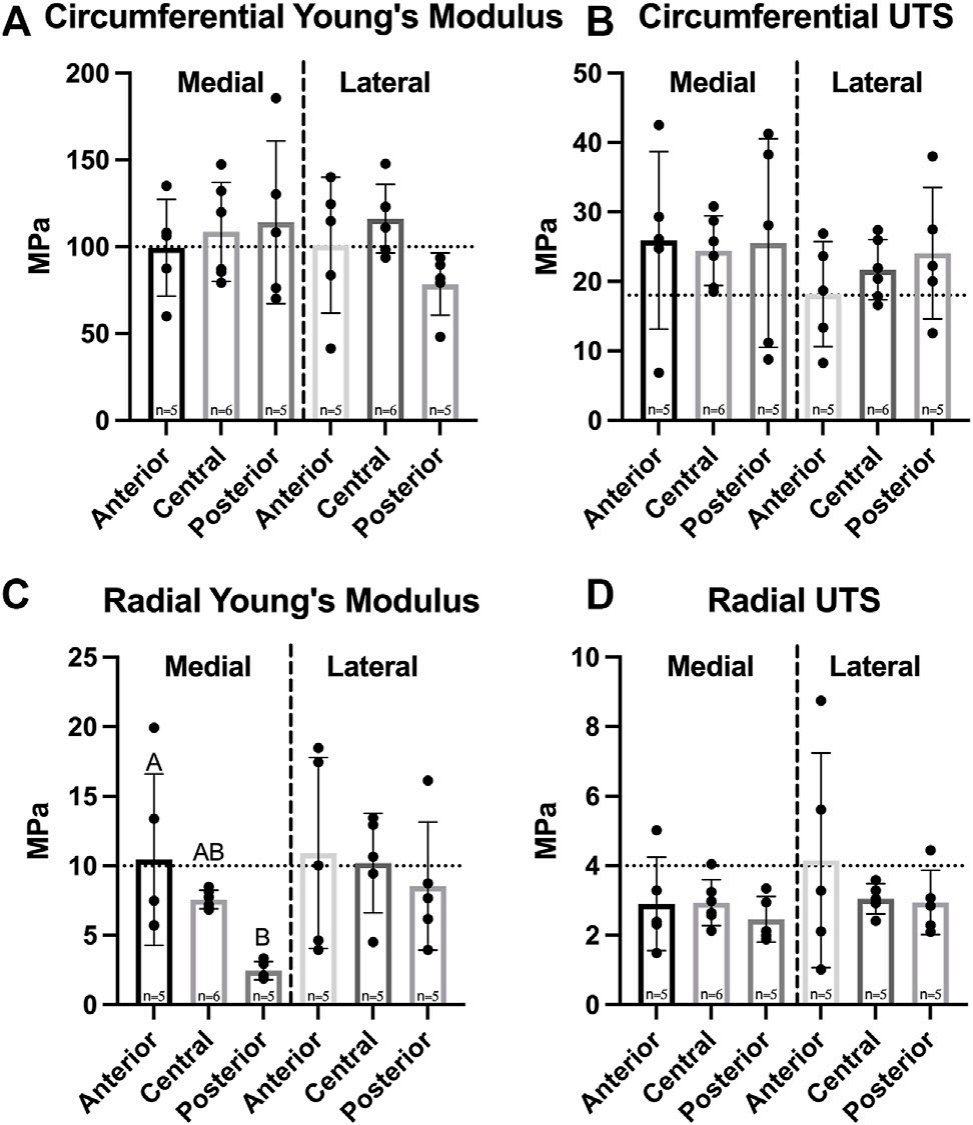
Tensile properties of Yucatan minipig knee menisci. **(A,B)** Young’s Modulus and UTS of medial and lateral menisci are shown for the circumferential and radial directions, respectively. No significant difference was seen in circumferential stiffness and strength; **(C,D)** radial stiffness in the anterior region of the medial meniscus was significantly higher than the posterior region, though no significant difference was seen in radial tensile strength. All data are presented as means ± standard deviations. Statistical significance is indicated by bars not sharing the same letters within the same meniscus; additionally, horizontal dotted lines on the Y-axis represent human meniscus values from the literature for comparison to the minipig (Tissakht and Ahmed, 1995; Makris et al., 2011).

Biomechanical data revealed no significant differences in circumferential Young’s modulus between medial and lateral menisci or among their regions, which ranged from 99.4 to 114.1 MPa in the medial meniscus and 78.4–116.2 MPa in the lateral meniscus (Figure 4A); additionally, circumferential UTS ranged from 18.2 to 25.9 MPa, though no significant difference among regions in either meniscus was shown (Figure 4B). Radial Young’s modulus was not significantly different between menisci and ranged from 2.5 to 10.9 MPa; however, the anterior region of the medial meniscus was significantly higher than the medial posterior region. No significant differences among regions in the lateral meniscus were observed (Figure 4C). UTS in the radial direction ranged from 2.5 to 4.2 MPa. There were no significant differences between menisci or among regions within either meniscus (Figure 4D).

**FIGURE 5:**
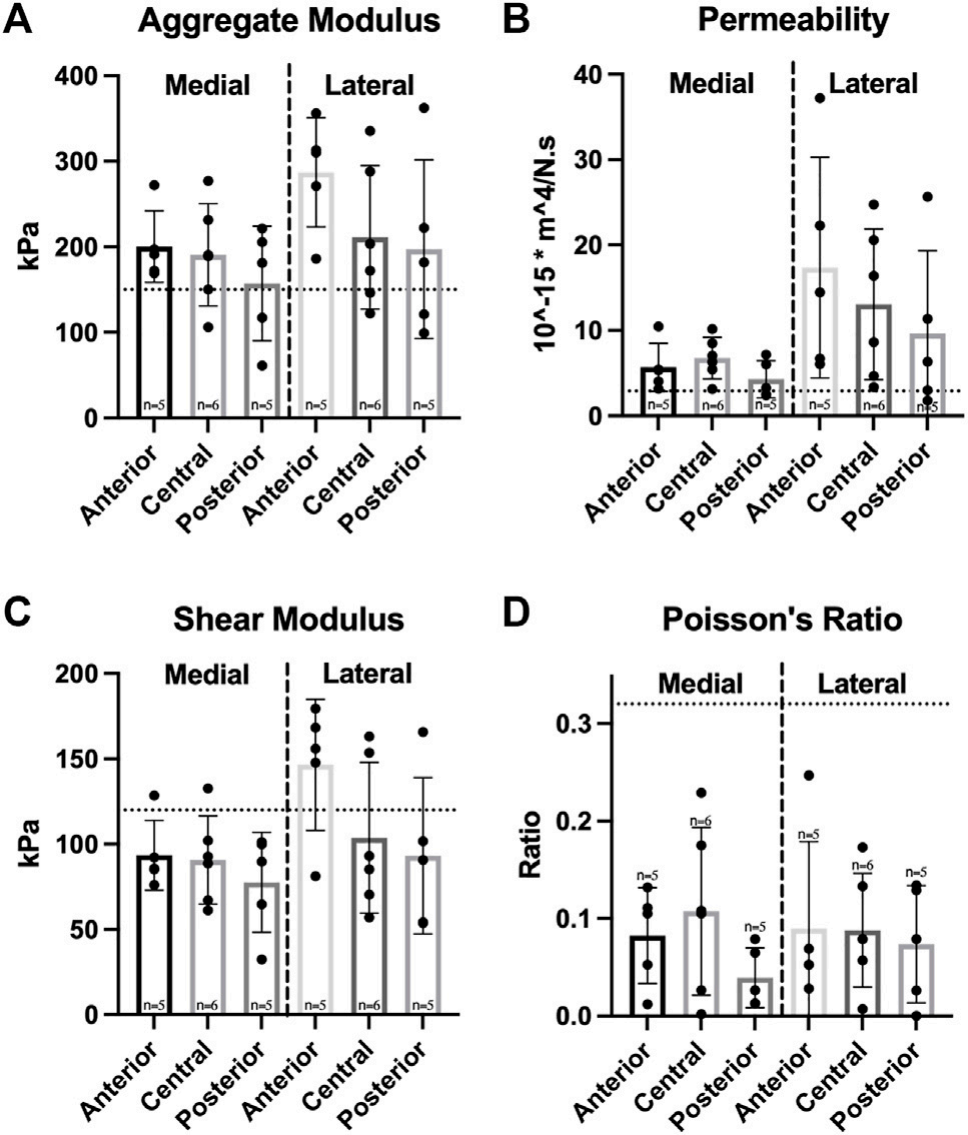
Compressive properties of Yucatan minipig knee menisci. **(A–D)** Aggregate modulus, permeability, shear modulus, and Poisson’s ratio are shown for medial and lateral menisci, respectively. Student’s *t*-test showed a significant difference between medial and lateral menisci in permeability values. One-factor ANOVA showed no significant differences in any compressive property among regions of the same meniscus. All data are presented as means ± standard deviations. Statistical significance is indicated by bars not sharing the same letters within the same meniscus; additionally, horizontal dotted lines on the Y-axis represent human meniscus values from the literature for comparison to the minipig (Sweigart et al., 2004; Makris et al., 2011; Morejon et al., 2021).

Compressive mechanical testing showed a significant difference in permeability values between medial and lateral menisci; however, no significant differences were observed among regions in either meniscus for the values of aggregate modulus, shear modulus, permeability, and Poisson’s ratio (Figure 5). Aggregate and shear modulus values ranged from 157 to 287 kPa and 91–147 kPa, respectively; both moduli trended highest in the anterior region of each meniscus and trended lowest in the posterior region.

### Tissue Biochemistry

**FIGURE 6:**
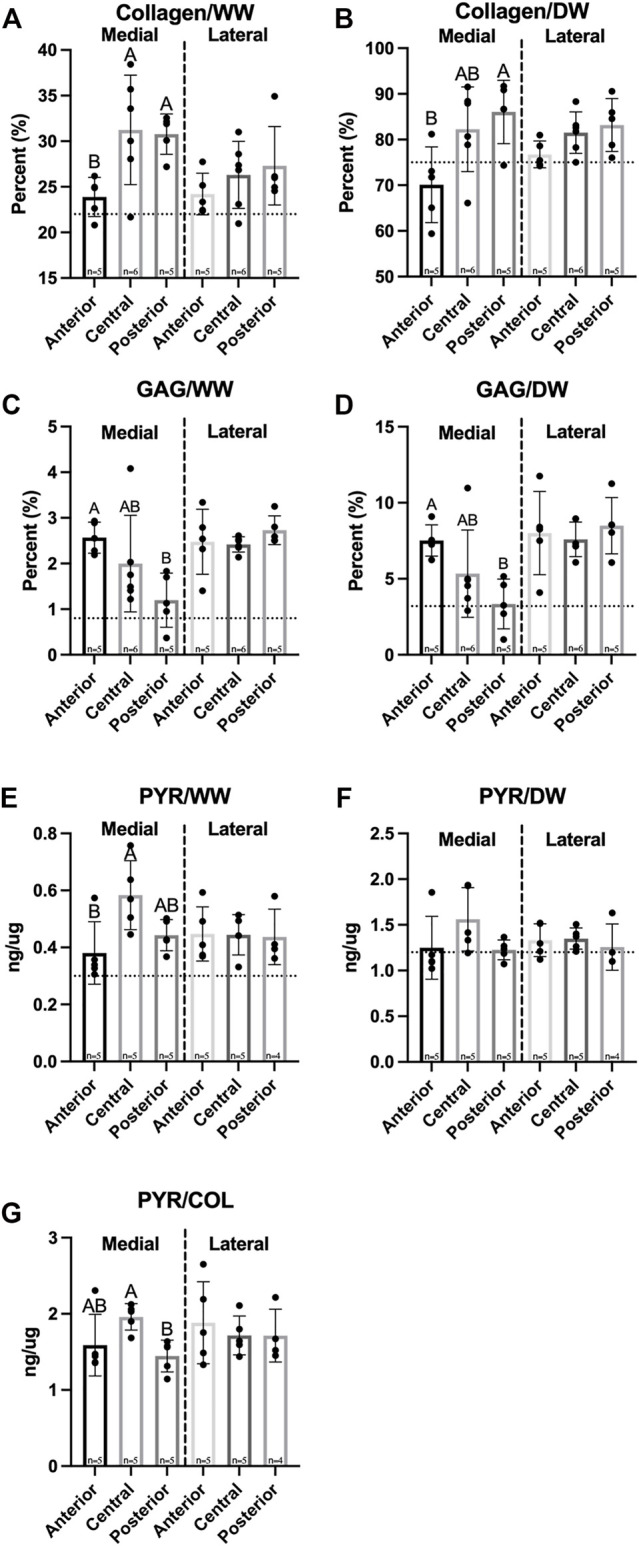
Biochemical properties of Yucatan minipig menisci. **(A–F)** Collagen, GAG, and pyridinoline crosslink content are shown normalized to wet and dry weights, respectively, in addition to **(G)** crosslinks normalized to collagen content. Student’s *t*-test shows significant differences between medial and lateral menisci in GAG content. One-factor ANOVA showed pyridinoline crosslinks normalized to collagen content was significantly higher in the central region of the medial meniscus compared to its posterior region; no significant differences were seen among regions in the lateral meniscus. All data are presented as means ± standard deviations. Statistical significance is indicated by bars not sharing the same letters within the same meniscus; additionally, horizontal dotted lines on the Y-axis represent human meniscus values from the literature for comparison to the minipig (Herwig et al., 1984; Takahashi et al., 1998; Makris et al., 2011).

A significant difference in hydration percentages was observed between medial and lateral menisci, which ranged from 64.0–67.8% (Table 1). Biochemical analysis showed collagen (COL) and GAG throughout both menisci, with concentrations per wet weight (WW) ranging from 23.9–31.3% COL/WW and 1.20–2.57% GAG/WW, respectively (Figures 6A,C). There were no significant differences between menisci in collagen content normalized to wet weight or dry weight (DW). Significantly less COL/WW was observed in the anterior region of the medial meniscus compared to its other regions, while no significant differences among regions in the lateral meniscus were observed. COL/DW in the medial meniscus was significantly higher in the posterior region compared to the anterior; no significant differences in COL/DW were found among regions in the lateral meniscus (Figure 6B). Significant differences between menisci were observed for GAG/WW and GAG/DW. In the medial meniscus, the anterior region had significantly more GAG/WW and GAG/DW than the posterior region; no significant differences in GAG/WW or GAG/DW were seen among regions in the lateral meniscus (Figures 6C,D).

Pyridinoline (PYR) crosslink content normalized to WW was not significantly different between medial and lateral menisci and ranged from 0.38 to 0.58 ng/µg. The central region of the medial meniscus contained significantly more PYR/WW compared to the anterior; there were no significant differences in PYR/WW content among lateral meniscus regions (Figure 6E). In addition, there were no significant differences in PYR/DW between menisci or among their regions (Figure 6F). Finally, PYR/COL ranged from 1.45 to 1.96 ng/µg and was not significantly different between medial and lateral menisci (Figure 6G).

### Anisotropy

**FIGURE 7:**
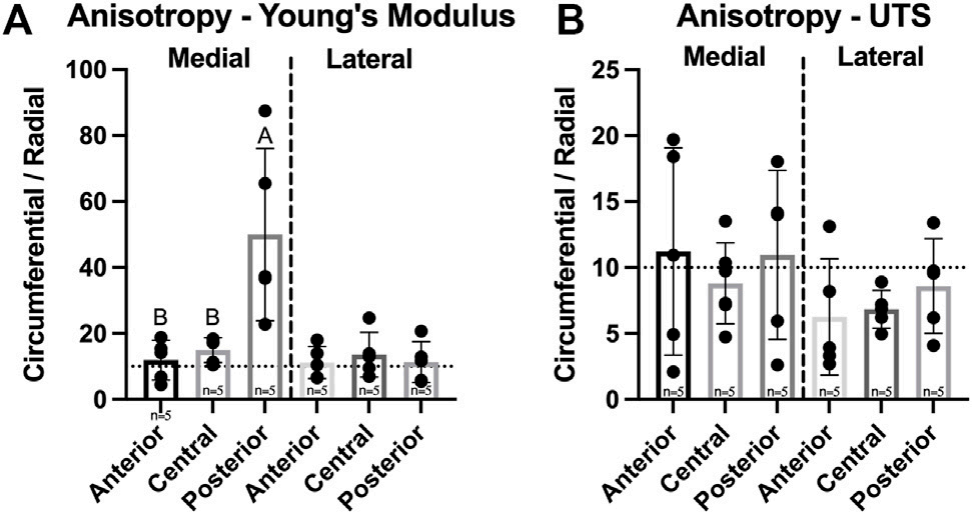
Anisotropy index of Yucatan minipig meniscus tensile properties. **(A,B)** Anisotropy indices are shown for tensile Young’s modulus and UTS, respectively. Student’s t-test showed a significant difference in Young’s modulus anisotropy values between menisci. One-factor ANOVA showed the posterior region of the medial meniscus was significantly more anisotropic in tensile stiffness than its other regions, while no significant differences were seen among regions of the lateral meniscus. All data are presented as means ± standard deviations. Statistical significance is indicated by bars not sharing the same letters within the same meniscus; additionally, horizontal dotted lines on the Y-axis represent human meniscus values from the literature for comparison to the minipig (Makris et al., 2011).

For the assessment of anisotropy, tensile properties of each region in both medial and lateral menisci were collected from two testing directions–circumferential and radial. Circumferential values were then divided by radial values to produce an anisotropy index. A significant difference between medial and lateral menisci was observed for tensile Young’s modulus but not for UTS. The Young’s modulus anisotropy index ranged from 11.2–49.9 and was significantly different among regions in the medial meniscus; the posterior region of the medial meniscus was significantly higher than other regions in the medial meniscus, while there were no significant differences among regions in the lateral meniscus (Figure 7A). UTS anisotropy levels ranged from 6.3–11.2 and no significant differences between menisci or among regions in either meniscus were found (Figure 7B).

## Discussion

The objective of this study was to characterize the knee menisci of Yucatan minipigs because the minipig has been proposed as a large animal model for translational cartilage and meniscus research. This was performed through an extensive analysis of structure-function relationships within the native tissue by region, which was motivated by known regional differences in human menisci. The data may provide design criteria to tissue engineers who aim to create repair and replacement technologies for the knee meniscus and to researchers that aim to test novel meniscal technologies in large animals. Notably, previously unexplored characteristics, such as the degree of collagen crosslinking within minipig menisci, were elucidated using an LC-MS assay. With regard to the hypothesis that gross morphological properties would be comparable to human menisci ranges, it was found that the regional width and peripheral height of minipig menisci fell within human ranges. Additional hypotheses that regional differences in mechanical properties would be observed and that regional differences in mechanical properties would correspond to differences in structural characteristics were also supported by the data. Support for these hypotheses is significant because the data imply that analogous products designed for human menisci would likely be functional in the minipig, allowing for human meniscal products to be tested in this animal.

Morphological features of the Yucatan minipig menisci were measured to assess the similarity between native minipig and human tissue; morphologically similar tissues between species could allow for translation of surgical techniques in addition to engineered meniscus implants. This study found minipig menisci to be comparable to human menisci, which measure 33.3–39.8 mm in anteroposterior length, 8.5–14.8 mm in peripheral height, and 5–7 mm in regional width, respectively. For example, values measured for the minipig menisci dimensions were within ranges seen in human menisci for 2 out of 3 properties - average peripheral height and average regional width (Erbagci et al., 2004; Takroni et al., 2016); the lateral minipig meniscus trended higher in anteroposterior length than the medial meniscus and is approximately 28% shorter in length than the lateral human meniscus (Yoon et al., 2011). Despite this difference, minipig anteroposterior lengths are comparable to other animal models that have been used in knee meniscus research such as sheep, goats, and farm pigs that measure 22–26 mm on average (Proffen et al., 2012; Brzezinski et al., 2017). Additionally, the minipig and human both exhibit higher peripheral height values in the lateral meniscus compared to the medial side (Table 1). The posterior regions of both minipig menisci were significantly wider than their respective anterior and central regions, similar to human menisci; the posterior region of the lateral minipig meniscus, the widest by average in this study, was only 2% smaller than the average width reported for the lateral meniscus in the human. Additionally, comparable to what is seen in humans (Chevrier et al., 2009; Fedje-Johnston et al., 2021), histology of minipig meniscus cross sections showed a collagen network throughout the tissue, a positive staining for GAG, and cells dispersed throughout the tissue (Figure 3). Overall, minipig knee menisci provide gross morphological similarities to humans in terms of their peripheral height and regional width, which could allow for the ready implantation and, eventually, translation of engineered tissues for their repair or replacement.

The knee meniscus functions by developing tension when under compressive load, highlighting the importance of both mechanical properties for the meniscus. It was found that there were no significant differences in tensile stiffness and strength in the circumferential or radial directions between medial and lateral menisci (Figure 4). Additionally, no significant differences in circumferential tensile properties among meniscus regions were observed, replicating what is seen in humans; at their peak, minipig meniscus circumferential stiffness and strength are 81 and 138% of the peak values recorded in humans, respectively (Tissakht and Ahmed, 1995). Radial stiffness of minipig menisci were on par to those of human menisci; values averaged across both medial and lateral menisci and regions were 8.3 MPa for the minipig and 10.8 MPa for humans (Tissakht and Ahmed, 1995) (Figure 4C). In terms of compressive properties, only permeability was significantly different between medial and lateral menisci; this difference between menisci was not seen in human tissue in a study that measured compressive properties using stress-relaxation (Morejon et al., 2021). Additionally, the homogeneity seen among regions in Yucatan minipig menisci (Figure 5) is not reflected in the human, albeit a similar trend was identified; the anterior region of the human medial meniscus is stiffer than its central and posterior regions, and is 80% as stiff as the anterior region of the medial meniscus in the Yucatan minipig (Sweigart et al., 2004). Biomechanical properties crucial to meniscus functionality, such as circumferential and radial tensile properties, were comparable between minipigs and humans; because of this, it is plausible that a meniscus implant with mechanical properties akin to those of human menisci can survive within the minipig knee environment during translational studies.

For humans, longitudinal tears occur more often in the medial posterior meniscus when compared to the anterior region (Skaggs et al., 1994). It has been suggested that the posterior region of the human medial meniscus bears more load than the anterior region and, thus, experiences larger radial stresses that lead to longitudinal tears (Ahmed and Burke, 1983). In the minipig, this study showed that the posterior region exhibited significantly lower radial stiffness than the anterior region (Figure 4C), which also corresponded to differences in composition (Figure 5). Thus, although there are currently no data on meniscal tears in minipigs, the data here would suggest that, with its lower mechanical properties, the minipig may share similarities with humans in having menisci that are more prone to injuries in the medial posterior region. The mechanical data obtained here may further be supported by differences in structure, such as the density or thickness of radially aligned collagen fibers (Skaggs et al., 1994), which warrant additional structural studies.

Because regional differences in mechanical properties of knee menisci have been identified in humans and other species such as cows, farm pigs, rabbits, and baboons (Skaggs et al., 1994; Tissakht and Ahmed, 1995; Sweigart et al., 2004), it is crucial to investigate the biochemical composition of minipig menisci toward understanding their mechanical function. Minipig and human menisci share similar levels of hydration, with 67.8% hydration in the anterior region of the minipig lateral meniscus being just under the literature value of 72% for human menisci (Makris et al., 2011). Collagen accounts for 23.9–31.3% per wet weight of minipig meniscus tissue (Figure 6B), and human menisci contains 22% COL/WW (Herwig et al., 1984). In terms of GAG content, values in the minipig meniscus reached as high as 2.73% GAG/WW (Figure 6C), which is approximately 3-times higher than in humans (Herwig et al., 1984). Notably, the posterior region of the medial meniscus contained significantly less GAG per wet and dry weights than the anterior region. Although there were no significant differences in compressive properties among regions in the medial meniscus, the posterior region had the lowest aggregate and shear moduli values. Overall, minipig meniscus collagen and GAG content were on par with or slightly exceeded levels seen in the human.

In addition to measuring collagen and GAG content, quantifying pyridinoline crosslinks is crucial to understanding the structure-function relationship of the minipig knee meniscus because these crosslinks have been shown to correlate with tensile properties of menisci and other collagenous tissues (Chan et al., 1998; Williamson et al., 2003; Eleswarapu et al., 2011). Pyridinoline crosslink content normalized to dry weight trended highest in the central region of the medial minipig meniscus and was measured at approximately 0.16%, which is higher than levels obtained in human menisci using an HPLC fluorescence detection assay at 0.12% (Takahashi et al., 1998). It should be noted that values in the present study were obtained using an LC-MS method, which has been shown to be more precise and accurate than HPLC fluorescence detection methods (Milićević et al., 2010; Wang et al., 2012; Bandeira et al., 2013; Bielajew et al., 2020, 2021). Pyridinoline crosslink content, for example, has been quantified using LC-MS techniques in bovine articular cartilage, showing crosslink levels of 0.12% of total dry weight, which were on par with values recorded in this study (Espinosa et al., 2021a). The posterior region of the medial meniscus contained significantly fewer crosslinks normalized to collagen content compared to the central region (Figure 6G), which may contribute to the low radial tensile stiffness in the posterior region. Overall, the medial meniscus contained regional variability in biochemical content while the lateral meniscus was more homogeneous throughout; this is reflected in the mechanical properties and anisotropy indices of the medial meniscus.

The anisotropic organization of ECM within the meniscus is crucial to the tissue’s function. Circumferential tensile stiffness and strength of menisci have been reported to be approximately 10-fold higher than those of the radial direction in many species (Fithian et al., 1990). Tensile anisotropy indices were also measured, defined as circumferential tensile properties normalized to those in the radial direction (Gonzalez-Leon et al., 2020), in this study for Yucatan minipig menisci tensile stiffness and strength. These ranged from 11.2–49.9 and 6.3–11.2, respectively, and were similar to those previously reported (Fithian et al., 1990). The medial meniscus however, contained a significantly higher anisotropy index for tensile stiffness in its posterior region compared to other regions (Figure 7A), likely stemming from the low tensile properties in the radial direction. It is worth noting, though, that radial tensile values in this region of the minipig were still on par with those reported for the same meniscal region in humans (Tissakht and Ahmed, 1995). In summary, the posterior region of the minipig meniscus, thus, has a higher degree of anisotropy, less crosslinked collagen, and lower radial tensile properties compared to other medial regions; these findings correspond to a region in the human medial meniscus where more injuries have been reported (Skaggs et al., 1994), showing the clinical relevance of using the minipig as a large animal model.

While this study elucidated that minipig menisci morphological, mechanical, and biochemical properties fall within native human tissue ranges, it is important to note that additional investigations into minipig meniscus properties could further validate these findings. Meniscus structure-function relationships have been shown to vary by zone (i.e., outer red-red zone versus inner white-white zone) in pigs and other species (Chevrier et al., 2009). Compressive properties and GAG content, for example, have been shown to be higher in the inner white-white zone of human and porcine menisci when compared to the outer red-red zone (Nakano et al., 1997; Scott et al., 1997; Tanaka et al., 1999); because this study collected biochemical samples from the middle white-red zone, additional studies are warranted to compare outer and inner zones. Furthermore, as this study utilized menisci from both male and female minipigs, sex-specific differences that may exist in meniscus properties were not able to be elucidated. Identifying sex-based differences for meniscus properties, should they exist, might allow for better understanding of meniscal function and pathophysiology in humans; fibrocartilages such as the TMJ disc, for example, have a higher frequency of injury in female patients when compared to male patients (Warren and Fried, 2001). Additionally, human meniscus characteristics such as GAG content have been shown to decrease with age (Clark and Ogden, 1983); investigation into minipig menisci at different stages of development could provide further insight into appropriate models to consider in preclinical research. These factors, investigated with an adequate number of experimental samples to generalize the findings, could thus provide crucial insight into minipig meniscus structure-function relationships.

The prevalence and economic impact of meniscal injuries motivate tissue engineers to create novel regenerative solutions. For these new implant technologies to successfully translate from the benchtop to the clinic they must first undergo extensive preclinical testing in a large animal model. It is crucial to find an appropriate animal model with similar structural, mechanical, and biochemical characteristics to humans and, ideally, a docile temperament to facilitate post-surgical care. Minipigs such as the Yucatan breed have been proposed as animal models for studies involving injuries to articular cartilage and the knee meniscus. Engineered meniscal implants should aim to recapitulate native tissue properties to increase their chances of survival in the native knee’s biomechanical environment. The characterization this study provides shows that the Yucatan minipig meniscus is comparable to humans in terms of morphological, mechanical, and biochemical properties. In addition, human meniscus injury patterns were considered when identifying an analogous location where they may occur in minipigs. These findings provide design criteria for tissue engineers that aim to create regenerative solutions to meniscal injuries and support use of the Yucatan minipig as a large animal model for translating meniscal therapies.

## Data Availability

The raw data supporting the conclusion of this article will be made available by the authors, without undue reservation.
